# The complete chloroplast genome of *Ananas comosus* var. *erectifolius* (L.B. Smith) Coppens & Leal

**DOI:** 10.1080/23802359.2022.2039081

**Published:** 2022-03-02

**Authors:** Chaoyang Liu, Wei Zhang, Yehua He

**Affiliations:** a Key Laboratory of Biology and Germplasm Enhancement of Horticultural Crops in South China, Ministry of Agriculture and Rural Affairs, Guangzhou, Guangdong, China; b College of Horticulture, South China Agricultural University, Guangzhou, Guangdong, China

**Keywords:** *Ananas comosus* var*. erectifolius*, Bromeliaceae, chloroplast genome, industrial application, phylogenetic analysis

## Abstract

*Ananas comosus* var. *erectifolius* (L.B. Smith) Coppens & Leal, a tropical plant from Bromeliaceae family, has immense applications, especially for fiber production of excellent quality. The lack of available chloroplast (cp) genome information limits its breeding and application. Here, we assembled its complete cp genome using Illumina high-throughput sequencing technology. The cp genome size is 159,983 bp, with 37.4% GC content, including a large single copy region (LSC) of 87,787 bp, a small single copy region (SSC) of 18,606 bp, and a pair of inverted repeat regions (IRs) of 26,795 bp. It encodes 89 protein-coding, 38 tRNA and 8 rRNA genes. Phylogenetic analysis showed that *A. comosus* var*. erectifolius* was close to *Ananas comosus*. The complete cp genome sequences could provide valuable information for variety breeding and genetic analysis of agronomic and economic traits in *A. comosus* var*. erectifolius.*

*Ananas comosus* var. *erectifolius* (L.B. Smith) Coppens & Leal, popularly known as curauá, is a plant species from the Bromeliaceae family in the order of Poales (Baker and Collins 1939). It is native to the Amazon region and distributed in wide parts of the world. Fiber produced from its leaves is one of the most resilient plant fibers and largely used in leather, synthetic and automobile industry (Mishra et al. 2004). This species is an environmentally friendly and biodegradable natural resource for industrial use, and also has significant social roles by using manpower of low-income populations (Barbosa et al. 2015). Its smooth and erect leaves are probably owing to the long term artificial selection for an abundance of long easily- extractable fibers. Apart from being a medicinal plant, the inflorescences of *A. comosus* var. *erectifolius* also present values for the production of cut-flowers and landscape-gardening (Leão et al. 2009; Teixeira et al. 2019; Manigandan et al. 2021). As a closely related plant species of pineapple, its special traits that are responsible for the valuable fiber production, like the dense, erect and smooth leaves, have the potential to be transferred into the commercial cultivars of pineapple through inter-specific hybridization, which might be helpful for enhancing the comprehensive utilization value of pineapple (Jawaid et al. 2020). Moreover, owing to the absence of spines and purplish leaves of *A. comosus* var. *erectifolius*, it can also be used as parent in the genetic population construction for the location of the spine/color-related genes. For its extensive application in the industry and other field, large amount of researches were aimed to improving the quality and production of *A. comosus* var. *erectifolius* (Pereira et al. 2008; Moreira et al. 2016). Organelle genomes have been commonly utilized in the researches on taxonomy, phylogeny and evolution (Yang et al. 2019; Wang et al. 2020). However, little is known about the chloroplast genome of *A. comosus* var. *erectifolius*, which affect its application and breeding processes. In this study, the complete chloroplast genome of *A. comosus* var. *erectifolius* was characterized and the phylogenetic relationships with other related species were analyzed, which were helpful for variety breeding and genetic analysis of agronomic and economic traits in *A. comosus* var. *erectifolius.*

The fresh leaves of *A. comosus* var. *erectifolius* were collected from Horticultural Germplasm Conservation Center of South China Agricultural University for breeding and research in Guangzhou, Guangdong Province, China (113°22'4″ N, 23°9'5″ E). A specimen was deposited at South China Agricultural University (Chaoyang Liu, email: chyliu5@163.com) under the voucher number SCAU20190725001. The total genomic DNA was extracted from about 100 mg fresh leaves using a modified CTAB method (Doyle and Doyle 1987). Paired-end Libraries with an average length of 350 bp were constructed by using the Nextera XT DNA Library Preparation Kit (Illumina, San Diego, CA), and then the libraries were sequenced on Illumina Novaseq 6000 platform (Shenzhen Huitong Biotechnology Co. Ltd). In total, 16,822,772 raw reads were obtained and edited by using NGS QC Tool kit (Patel and Jain 2012), 4.99 Gb clean data was assembled by using the *de novo* assembler SPAdes v.3.11.0 software, with a k-mer set of 93, 95, 97, 103, 105, 107 and 115 (Bankevich et al. 2012). The chloroplast genome of *Ananas comosus* (Accession number: AP014632.1） was used as the reference. Finally, the assembled complete cp genome was annotated by via PGA software (Qu et al. 2019). The complete chloroplast genome was submitted to GenBank with Accession no. MZ457322.

The total length of *A. comosus* var. *erectifolius* cp genome is 159,983 bp, with 37.4% GC content, comprising a pair of IRs 26,795 bp, separating the LSC region of 87,787 bp and SSC region of 18,606 bp. There were 135 genes, including protein-coding genes, 8 rRNA genes, and 38 tRNA genes. Among of them, 15 genes (*trn*K-UUU, *rps*16*, trnG*-GCC, *atp*F, *rpo*C1, *trn*L-UAA, *trn*V-UAC, *pet*B, *pet*D, *rpl*2, *ndh*B, *trn*I-GAU, *ycf*68, *trn*A-UGC, *ndh*A) contained one intron, two genes (*clp*P and *ycf*3) contained two introns, while one gene (*rps*12) underwent trans-splicing.

Phylogenetic analysis was performed based on complete cp genomes of *A. comosus* var. *erectifolius* and other 20 related species reported in the Commelinids clade, *Arabidopsis thaliana* as outgroup. The chloroplast genome sequences were aligned with MAFFT 7.407 with default parameters (Nakamura et al. 2018), and then the maximum-likelihood tree was constructed by IQ-TREE 1.6.12 with the parameter (-bb 1000) (Nguyen et al. 2015). All the *Ananas* species formed a monophyly. The *A. comosus* var. *erectifolius* was a basal species in the *Ananas* genus (Figure 1). In conclusion, the characterized cp genome sequence of *A. comosus* var. *erectifolius* provides a useful genetic resource for future phylogenetic identification and analysis of important agronomic and economic traits.

**Figure 1.**
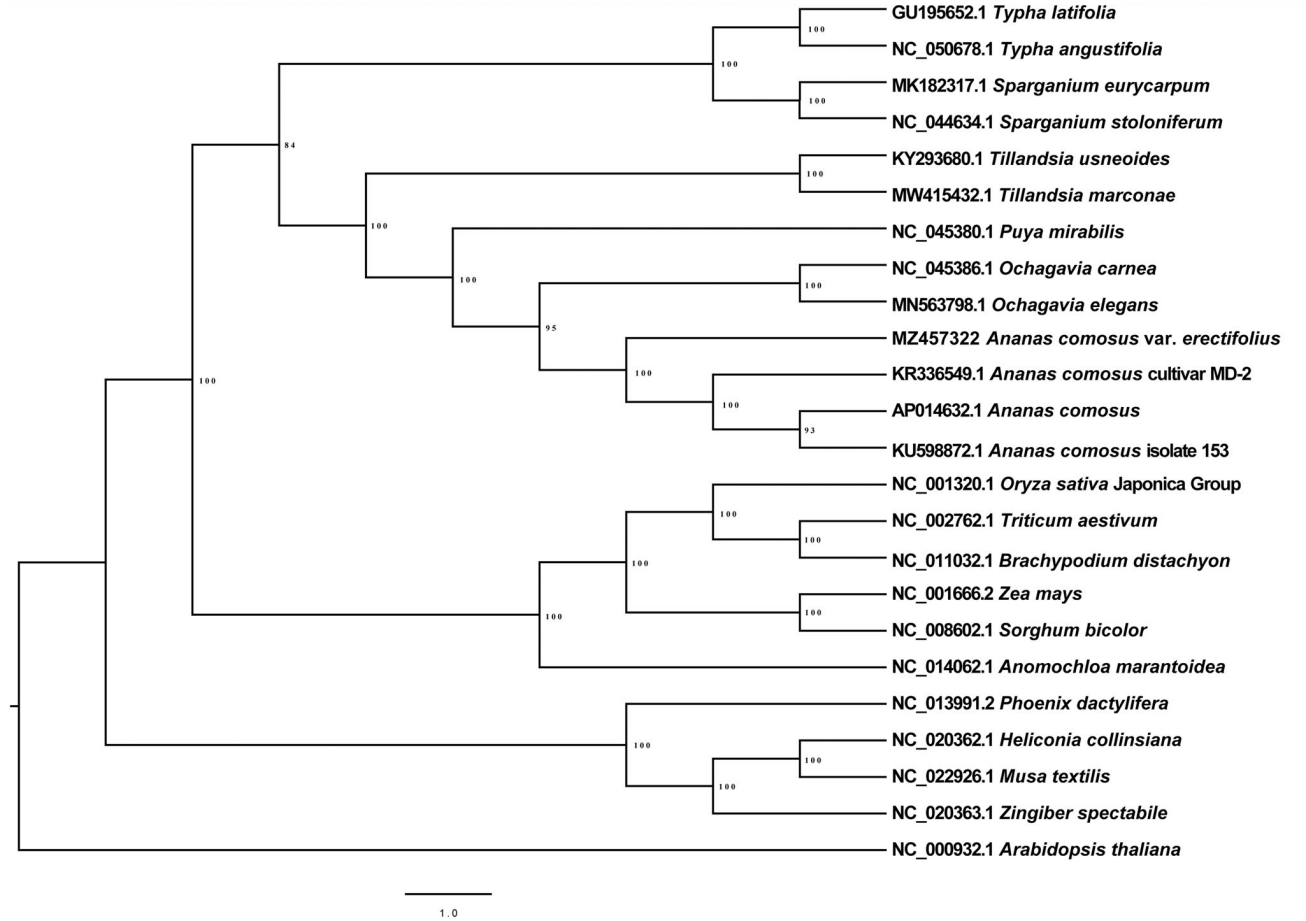
Maximum likelihood tree based on chloroplast genome sequences of the species in Commelinids, with *Arabidopsis thaliana* as outgroup. Numbers on each node are bootstrap support values from 1000 replicates.

## Data Availability

The genome sequence data that support the findings of this study are openly available in GenBank of NCBI at (https://www.ncbi.nlm.nih.gov/nuccore/MZ457322) under the accession no. MZ457322. The associated BioProject, SRA, and BioSample numbers are PRJNA742473, SRR14998994, and SAMN19955712 respectively.
